# Customizable Optical Force Sensor for Fast Prototyping and Cost-Effective Applications

**DOI:** 10.3390/s18020493

**Published:** 2018-02-07

**Authors:** Jorge A. Díez, José M. Catalán, Andrea Blanco, José V. García-Perez, Francisco J. Badesa, Nicolás Gacía-Aracil

**Affiliations:** 1 Departamento de Ingeniería de Sistemas y Automática, Universidad Miguel Hernández de Elche, 03202 Elche, Spain; jcatalan@umh.es (J.M.C.); ablanco@umh.es (A.B.); j.garciap@umh.es (J.V.G.-P.); nicolas.garcia@umh.es (N.G.-A.); 2 Departamento de Ingeniería en Automática Electrónica, Arquitectura y Redes de Computadores, Universidad de Cádiz, 11510 Puerto Real, Spain; javier.badesa@uca.es

**Keywords:** optical force sensor, printable sensor, cost-effective sensor, hysteresis correction, Prandtl–Ishlinskii model, perceptron

## Abstract

This paper presents the development of an optical force sensor architecture directed to prototyping and cost-effective applications, where the actual force requirements are still not well defined or the most suitable commercial technologies would highly increase the cost of the device. The working principle of this sensor consists of determining the displacement of a lens by measuring the distortion of a refracted light beam. This lens is attached to an elastic interface whose elastic constant is known, allowing the estimation of the force that disturbs the optical system. In order to satisfy the requirements of the design process in an inexpensive way, this sensor can be built by fast prototyping technologies and using non-optical grade elements. To deal with the imperfections of this kind of manufacturing procedures and materials, four fitting models are proposed to calibrate the implemented sensor. In order to validate the system, two different sensor implementations with measurement ranges of ±45 N and ±10 N are tested with the proposed models, comparing the resulting force estimation with respect to an industrial-grade load cell. Results show that all models can estimate the loads with an error of about 6% of the measurement range.

## 1. Introduction

### 1.1. Background

In the framework of the AIDE H2020 European Project [1], a hand exoskeleton has been developed as a part of the multimodal system that will assist disabled people during the realization of a wide range of activities of daily living [2]. This device must be able to pick up various everyday objects such as glasses, adapted cutlery or handles, bottles, dishes, or a toothbrush. Hence, the grip must be safe in order to provide real independence to the user. Additionally, a rehabilitation version of that device is being developed in parallel [3]. For both purposes, it is interesting to have a measurement of the interaction force between user and hand exoskeleton. This information could be useful from different points of view:

- Feedback in an impedance control system,
- Comparison with a threshold to limit the interaction force between user and device for safety reasons,
- Detection of movement intention,
- Measurements to develop objective clinical assessment systems.

### 1.2. State of the Art

In the current literature, we can find a wide diversity of sensor technologies that have been implemented in wearable and exoskeleton robots for the same purpose, such as torque sensors [4,5], strain gauges [6,7,8], miniature load cells [9,10,11], force-sensitive resistors [12,13,14], and Hall effect force sensors [15,16]. However, these technologies present some problems for its use in exoskeletons and wearable devices:

- In case of torque sensors, they measure the load in the motor shaft, which in over-constrained mechanisms implies not measuring all the interaction forces. Additionally, they are limited to be used with rotary actuators.
- The main shortcoming of strain gauges is the difficulty in their placement and fixation. Strain gauges must be glued firmly to a surface that deforms in a concrete way, resulting in relatively complex geometries or miniaturization hardly achievable without specialized material assets.
- There is a wide variety of miniature load cells; however, most of them can only measure compression forces, while compression–extension sensors may result unaffordable or not sufficiently miniaturized.
- Force-sensing resistors depend heavily on the surface contact and are not sufficiently reliable to be considered for this application.
- Other technologies like Hall effect force sensors may result in high-consumption and bulky solutions that can be integrated only in the biggest wearable devices.

Optical force sensors may suppose a promising alternative to overcome these shortcomings that make the force measurement in wearable devices difficult. The wide variety of optical and opto-electronical components commercially available allow multiple designs and optical arrangements that may be easily adapted to the requirements of these kinds of devices.

With optical technology, high precision and resolution can be achieved by using optical fiber sensors, which rely on determining the distortion produced in the fiber due to an external load. This strain may be measured with multiple techniques, such as Fabry–Perot interferometers [17,18] or Bragg gratings [19,20]. These solutions have potential applications in high performance applications and are not affected by the electromagnetic fields produced by actuators or medical instrumentation; however, they might result in being too expensive for most wearable applications.

More affordable solutions can be implemented by using light emitting diodes (LED) and photo-receivers that measure the incident light. The light can be guided with optical fibers to conserve the robustness to strong electromagnetic fields [21,22]. Other implementations work by direct interaction between the emitter and receiver, without any intermediate optical element [23,24,25] resulting in miniaturized devices. The addition of intermediate optical components can increment the design flexibility with a sacrifice of miniaturization capability [26,27,28].

### 1.3. Objectives

The goal of the presented research consists in developing the architecture of a force sensor with compression–extension measurement capability, which might be built completely with fast-prototyping techniques and materials, so that it can be easily shaped in a custom form to be integrated into a design still in the prototype stage. Additionally, to make its use more flexible, the proposed sensor architecture is also designed to allow the adjustment of its performance once the sensor is built and placed by only replacing some simple elements.

Despite the design being intended to be as general-purpose as possible, one of the short-term goals of the research is to obtain a device that can be integrated into a small wearable device, concretely the hand-exoskeleton mentioned in Section 1.1.

## 2. Materials and Methods

### 2.1. Hardware Description

**Figure 1:**
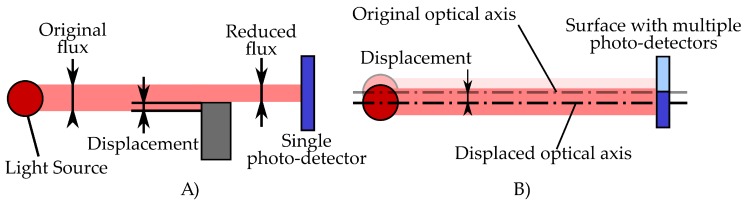
Optical micrometric sensors strategies. (**A**) light incidence is modified by occlusion of the photo-detector. (**B**) displacement is measured by modifying the position of the focus point in a matrix of photo-detectors.

The proposed architecture consisted of optical sensors based on the use of photo-detectors. These kinds of sensors can measure micrometric displacements by sensing fluctuations in the incidence of a light beam over a photo-detector. These variations in the light flux may be induced by occlusion of the light inciding on a single photo-detector (Figure 1, Left) or by modifying the position of the light focus over a segmented detector (Figure 1, Right).

This working principle allows the measurement of small displacements. Thus, if the measured displacement corresponds to a deformation of an elastic material, this geometrical distortion may be easily related to a stress and thus a force producing it.

Hence, a properly designed optical system in conjunction with a correctly sized deformable elastic structure may allow the measurement of forces with a concrete measurement range and sensitivity.

#### 2.1.1. Optical Architecture

Starting from the two methods to measure light fluctuations exposed above, it can be concluded that, in order to measure small displacements, the occlusion method would require a photo-detector with a high sensitivity since variation of the light flux is directly proportional to the displacement. However, the strategy based on the modification of the focus point in the light sensitive surface allows the addition of optical elements that can refract the light beam, amplifying the induced deviation and resulting in an increase of the sensitivity of the system.

**Figure 2:**
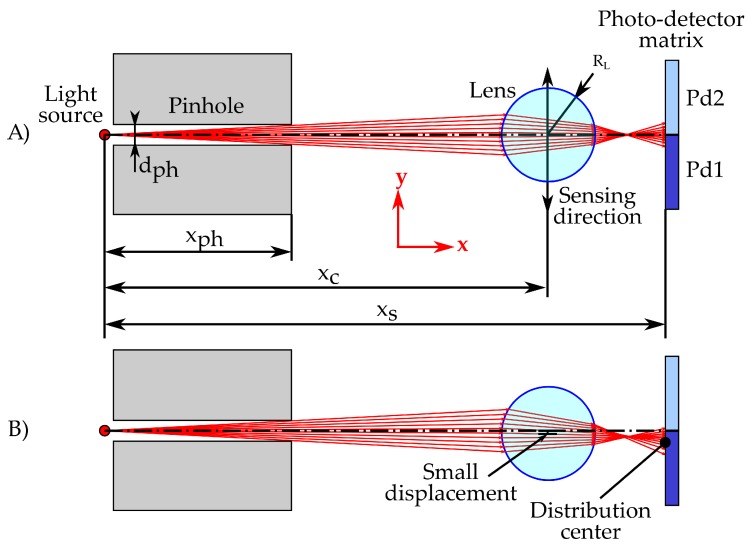
(**A**) proposed optical architecture in nominal position (perfect alignment) and geometrical parameters that determine the sensor response. (**B**) resulting light distribution in the photo-detecting surface after applying a small displacement in the lens in the *y*-direction (direction of the force to be measured).

In particular, the proposed optical assembly, shown in Figure 2, consists of:

- A point light source, which emits a directionless light front.
- A pinhole to narrow the light front, reducing the stray light and obtaining the light beam.
- A lens that refracts the light beam and collimates it over the light sensitive surface. This element will be the element subdued to the displacement to be measured. It will have a focal length short enough to induce a high deviation of the light beam when a small misalignment is applied (Figure 2, Bottom).
- A light sensing surface, composed of a matrix of photo-detectors so the position in which the light is focused can be determined by the difference of the light measured in each element of the matrix.

This optical scheme has five geometrical design parameters, corresponding to the distance of the pinhole output ($x_{ph}$), its diameter ($d_{ph}$), the distance of the lens center ($x_{c}$), and the distance of the photo-sensitive surface ($x_{s}$), all of them measured from the center of the light source. The refractive index of the lens is an additional parameter that will determine the response of the system. However, since it depends on the material constituting the lens, only a very limited set of values can be chosen.

In order to achieve a system with the desired behavior, a theoretical model has been constructed, in order to be able to check which parameter configurations fit better with the intended performance. Since the focal length of the lens has the same magnitude order than the rest of the dimensions, the paraxial approximation cannot be applied to this system. Thus, a 2D ray-tracing model [29] has been stated to simulate the path followed by a certain number of rays from the light source to the photo-sensitive surface:

1. First of all, a set of $q_{rays}$ rays are generated from the light-source-pinhole assembly. Each ray (*i*) is defined by its origin ($x_{i}^{o}$, $y_{i}^{o}$) and the direction of propagation expressed by the angle $\alpha_{i}$, measured clockwise from the optical axis. These parameters are computed using the Equations (1) to (3):
  (1) $$\begin{array}{cc} x_{i}^{o} & =0, \end{array}$$
  (2) $$\begin{array}{cc} y_{i}^{o} & =0, \end{array}$$
  (3) $$\begin{array}{cc} \alpha_{i} & =\left(i-\frac{q_{rays}}{2}\right)atan\left(\frac{d_{ph}}{2x_{ph}}\right). \end{array}$$
2. Once generated, the first intersection ($x_{i}^{r}$,$y_{i}^{r}$) between each ray and the spherical lens is computed solving the Equations (4) and (5). In each intersection, the normal vector to the surface (${\vec{u}}_{i}$) and a vector (${\vec{n}}_{i}^{in}$) with the same direction than the incident ray and modulus equal to the refractive index ($n^{in}$) of the propagation medium are computed using Equations (6) and (7):
  (4) $$\begin{array}{cc} R_{L}^{2} & ={\left(x_{i}^{r}-x_{c}\right)}^{2}+{\left(tan\left(\alpha_{i}\right)\left(x_{i}^{r}-x_{i}^{o}\right)+y_{i}^{o}-y_{c}\right)}^{2}, \end{array}$$
  (5) $$\begin{array}{cc} y_{i}^{r} & =tan\left(\alpha_{i}\right)\left(x_{i}^{r}-x_{i}^{o}\right)+y_{i}^{o}, \end{array}$$
  (6) $$\begin{array}{cc} {\vec{u}}_{i} & =(x_{i}^{r}-x_{c},y_{i}^{r}-y_{c}), \end{array}$$
  (7) $$\begin{array}{cc} {\vec{n}}_{i}^{in} & =n^{in}\frac{\left(x_{i}^{r}-x_{i}^{o},y_{i}^{r}-y_{i}^{o}\right)}{\sqrt{{\left(x_{i}^{r}-x_{i}^{o}\right)}^{2}+{\left(y_{i}^{r}-y_{i}^{o}\right)}^{2}}}. \end{array}$$
3. The refraction of the rays is modeled by applying the Snell’s law in its vectorial form (Equations (8) and (9)), to obtain the vector ${\vec{n}}_{i}^{out}$, which has the same direction than the refracted ray and a modulus equal to the refractive index of the new propagation medium ($n_{i}^{out}$):
  (8) $$\begin{array}{cc} \left({\vec{n}}_{i}^{in}\times {\vec{u}}_{i}\right)\cdot \left({\vec{n}}_{i}^{out}\times {\vec{u}}_{i}\right) & =n_{i}^{in}n_{i}^{out}cos\left(\theta \right), \end{array}$$
  (9) $$\begin{array}{cc} |{\vec{n}}^{in}\times {\vec{u}}_{i}| & =|{\vec{n}}^{out}\times {\vec{u}}_{i}|. \end{array}$$
4. Steps 2 and 3 solve the refraction of a light beam (composed by multiple rays) that interacts with an arbitrary surface that separates two mediums with different refractive indexes. This procedure generates a new set of rays, which can be described using the Equations (10) to (12), by their origin ($x_{i}^{\prime o}$, $y_{i}^{\prime o}$) and the angle that defines the direction of propagation ($\alpha_{i}^{\prime }$), similarly to the original set:
  (10) $$\begin{array}{cc} x_{i}^{\prime o} & =x_{i}^{r}, \end{array}$$
  (11) $$\begin{array}{cc} y_{i}^{\prime o} & =y_{i}^{r}, \end{array}$$
  (12) $$\begin{array}{cc} \alpha_{i}^{\prime } & =acos\left(\frac{{\vec{n}}_{i}^{out}\cdot {\vec{u}}_{i}}{|{\vec{n}}_{i}^{out}\left|\right|{\vec{u}}_{i}|}\right). \end{array}$$
5. Steps 2 to 4 are performed again with the new refracted beam in order to compute the second refraction, which happens when it crosses the interface that separates the lens medium and the air. The resulting set of rays ($x_{i}^{\prime \prime o}$, $y_{i}^{\prime \prime o}$, $\alpha_{i}^{\prime \prime }$) corresponds to the beam focused by the lens.
6. Finally, the intersection of each resulting ray with the photo-sensitive surface ($y_{i}^{sensor}$) is determined by Equation (13). The individual photo-detector excited by each ray can be determined by the computed coordinate $y_{i}^{sensor}$, so the histogram of the number of rays per each photo-cell can be easily obtained:
  (13) $$\begin{array}{c} y_{i}^{sensor}=tan\left(\alpha_{i}^{\prime \prime }\right)\left(x_{s}-x_{i}^{\prime \prime o}\right)+y_{i}^{\prime \prime o}. \end{array}$$

One point that must be underlined is that, during the execution of the described algorithm, some equation systems can lead to multiple solutions. The relevant ones can be easily isolated by applying conditional sentences. Additionally, the Snell’s law in vectorial form also returns solutions corresponding to reflections, which have been neglected since this effect is expected to have low influence in the results.

This mathematical model allows the execution of simulations, by just evaluating it with the same input parameters ($x_{ph}$, $x_{c}$, $x_{s}$, $d_{p}h$, $R_{L}$, $q_{rays}$, $n^{air}$ and $n^{lens}$) varying the position of the lens ($y_{c}$). The resulting histograms may help to estimate the performance of the system for different input configurations.

Since the response of many photo-detectors is usually given in terms of incident light power, the number of rays that fall upon each photo-cell can be translated into these terms by just dividing the total power that passes throughout the pinhole ($P^{ph}$) by the number of computed rays. Thus, the power ($P_{j}^{pd}$) that receives each photo-cell (*j*) of the photo-sensitive surface can be computed by knowing the number of rays that falls on it ($q_{rays}^{i}$) as shown in Equation (14). Knowing the responsitivity (*r*) of the photo-detector, this power can be easily translated to a current. Furthermore, with a proper transimpedance and amplifying circuitry (with conversion factor *A*), a voltage value for each photo-detector can be obtained (Equation (15)):

(14) $$\begin{array}{c} P_{j}^{pd}=\frac{P^{ph}}{q_{rays}}q_{rays}^{i}, \end{array}$$

(15) $$\begin{array}{c} V_{j}^{pd}=ArP_{j}^{pd}. \end{array}$$

Summing up, with the optical arrangement shown in Figure 2, it is possible to estimate the displacement ($y_{c}$) of the lens as a function of the difference of electric response of a photo-sensitive electronic device (Equation (16)). This expression may be determined by applying a polynomial fitting to the results of this simulation method for several $y_{c}$ values:

(16) $$\begin{array}{c} y_{c}=f\left(V_{2}^{pd}-V_{1}^{pd}\right). \end{array}$$

**Figure 3:**
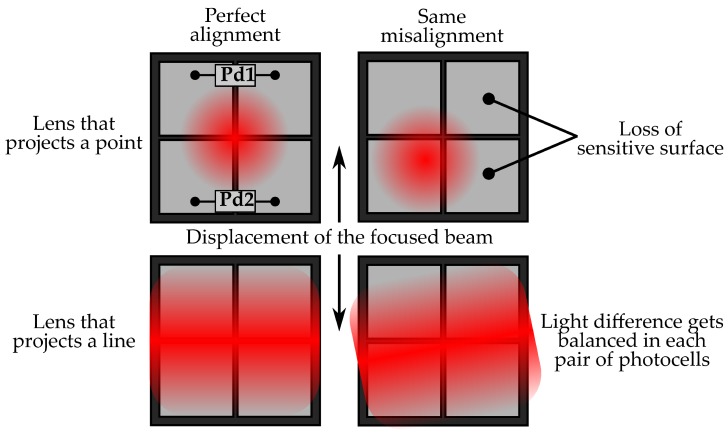
Scheme that shows the light focused by a lens in a 2 × 2 photo-detector matrix and the effect that the misalignment of the optical train produces in the light distribution.

To increase the robustness of the optical system to misalignment, a cylindrical geometry has been chosen for the lens so that it will project the point light source as a diffused line in the photo-detector matrix. This fact together with the two-dimensional distribution of photo-cells will allow for stacking the information of each row to achieve a better signal-noise ratio (SNR) and a more uniform light distribution that can balance optical deviations, as shown in Figure 3.

#### 2.1.2. Elastic Frame

Once the relation between the displacement of the lens ($y_{c}$) and the response of the photo-detector is known, it is necessary to relate this shift with the force that is going to be measured.

For this purpose, the proposed solutions consist of attaching the lens to an elastic element with a very high stiffness in all directions of movement and rotations except in that one in which the force wants to be measured. In this last direction, the type of elastic element should allow a fine tuning of the stiffness, so the behavior of the sensor can be adapted to the working conditions.

The most simple architecture would be the use of helical springs or elastic washers. The former solution has the inconvenience that helical springs should be guided in order to avoid displacements in other directions than the desired one, resulting in complex and not-compact structures, additionally, the commercially available range of stiffness and sizes might not be suitable for the desired application. Elastic washers might be a smaller solution; however, the range of possible stiffness is limited.

**Figure 4:**
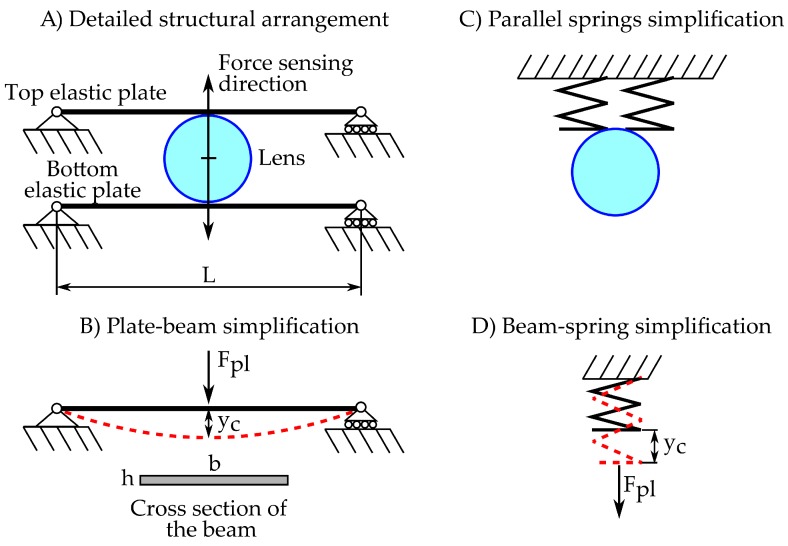
(**A**) proposed architecture to associate the lens displacement with the force to be measured; (**B**) beam to be solved to estimate the behavior of the elastic plate and the beam’s cross section; (**C**) both plates can be simplified to a parallel spring association; (**D**) spring equivalent to the beam shown in B.

The proposed arrangement achieves a balance between flexibility and ease of design and size. It consists of attaching the lens between two beam-mounted parallel elastic plates as shown in Figure 4A. Plates have a dimension much smaller than the other two, so they have a preferential direction of deformation, especially when subdued to bending actions. Thus, they do not require any kind of guidance to assure that the displacement of the lens is performed in the desired direction. However, a plate does not have enough stiffness when a torsion moment is applied, so a not-perfectly centered force could cause a non-desired rotation of the lens. This issue is solved by adding a second plate parallel to it, so the stiffness is highly increased, especially when the distance between plates has the same (or higher) order of magnitude than the big dimensions of the plate.

The deformation of a plate is governed by complex equations, which, in most cases, cannot be analytically solved, so numerical methods must be used. Since it is expected that countless imperfections stem from the fabrication process, the authors do not consider that a complex solving method would necessarily produce accurate results. Instead of this, the classical beam theory would be used to model the elastic behavior of the plates, which can be solved by analytic means and leads to handy expressions useful for sizing process. Hence, each plate will be solved as a beam with the cross section shown in Figure 4B.

In particular, the deformation of the center of the beam, which can be considered equal to the studied displacement of the lens ($y_{c}$), can be computed as shown in the Equation (17), where *L*, *b* and *h* are the dimensions specified in the Figure 4, *E* is the Young’s modulus of plate material and $F_{pl}$ the part of the total force that deforms that plate. Grouping the geometrical and material parameters in a constant, the beam can be simplified to a spring (Figure 4D) with the stiffness ($k_{pl}$) described in Equation (18). Since both plates are attached to the sensor’s frame and deform in the same direction, the equivalent springs can be associated in parallel as shown in Figure 4C. Thus, the relation between the force to be measured and the displacement of the lens can be computed by applying the one-dimensional form of Hooke’s law (Equation (19)):

(17) $$\begin{array}{cc} y_{c} & =\frac{L^{3}}{48Ebh^{3}}F_{pl}, \end{array}$$

(18) $$\begin{array}{cc} k_{pl} & =\frac{48Ebh^{3}}{L^{3}}, \end{array}$$

(19) $$\begin{array}{cc} F & =2k_{pl}y_{c}. \end{array}$$

As can be concluded by the Equations (17) to (19), for a determinate optical arrangement, multiple sensing ranges and sensitivities can be achieved by just changing the geometrical parameters of the plate. Special attention must be paid in the effect of the thickness (*h*). Since stiffness presents a cubic dependence on it, small changes lead to great variations in the stiffness, allowing a fast tuning of the range of measurement. Conversely, the width of the plate (*b*) has a proportional relation with the spring constant, so a fine adjustment of the sensor’s performance can be achieved with it. The length of the plate (*L*) does also have an important effect on the response of the system; however, its modification is discouraged since it normally implies a modification in the dimensions of the optical system, with hardly-predictable results.

#### 2.1.3. Manufacturing Process

One of the main objectives of this research consists of achieving a sensor, which can be mainly built with prototyping technologies, so its manufacture can be completely carried out in a research laboratory.

Regarding the optical system, all the required photo-electronic devices can be easily handled without special machinery or requirements. The light source can be implemented with a simple board holding a LED sensor, a simple voltage divider and two supply terminals. The photo-sensitive detectors may present different manufacturing difficulties according to the chosen technology. Hence, the authors propose the use of a photo-diode matrix with common-cathode arrangement so that only individual anodes and the common-cathode need to be outputted. These electronic boards can be easily machined in a small numerical control milling machine and soldered manually.

As for the lens that focuses the light beam, optical grade elements normally require a careful handling and special tools in order not to damage its optical properties. Thus, instead of using these kinds of components, a general purpose acrylic (PMMA) glass is cut to obtain custom-length cylindrical lenses. PMMA rods are an inexpensive industrial supply that can be easily found in a wide variety of diameters ranging from about 1 mm to several centimeters, providing a broad range of possible focal lengths. Since the used material is not optical-grade, properties such as refractive index or surface quality may present flaws. However, the desired feature of this architecture is the capability of working in such coarse conditions.

In respect of the frame of the sensor and the elastic elements that define the displacement of the lens, they are designed to be built by fused-filament 3D-printing process with the most extended materials used in this technology, i.e., Polylactic acid (PLA) and Acrylonitrile butadiene styrene (ABS). The most affordable 3D printers do not guarantee uniform and repeatable mechanical properties of the print since these depend on the printing parameters as well as the different imperfections that might appear due to the chemical composition of the material and dimmensional tolerances [30]. However, with a suitable oversizing, printed parts can work in strain–stress conditions far from the limit points. This is especially suitable when designing the rigid frame that places each element such as light source, photo-detector and plate supports.

As for the elastic plates, they also can be 3D printed under certain conditions. Concretely, they must be printed with layers parallel to the plate plane and complete infill. A conservative piece of criteria to choose an adequate size for plates of PLA (considering a Young’s modulus *E* = 2000 N/mm${}^{2}$ [30]) composition consists in seeking that the required displacement ($y_{c}$) do not exceed half the width of the individual plates. When this criteria cannot be achieved and the required plate width must be lower than the displacement, other materials can be used for building the plates. During this research, the possibility of using leaf springs made of AISI 301 Steel (*E* = 20,000 N/mm${}^{2}$) has been studied to produce a very sensitive sensor for a reduced range of measurement.

#### 2.1.4. Particular Implementation

As exposed in Section 1.3, this architecture was devised in order to obtain a sensor that could be easily integrated inside the frame of a hand exoskeleton. Thus, the prototype implemented to check its feasibility and performance is aimed at satisfying the requirements and loads of this application, concretely a range of measurement of ±40 N should be suitable for measuring the interaction between the exoskeleton and the hand. A more detailed description of the problem can be found in [31].

**Figure 5:**
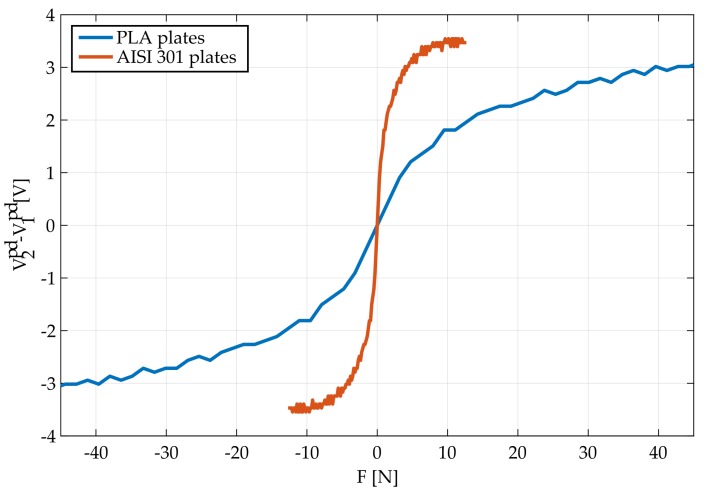
Computed response for the implemented sensor according to the kind of elastic plates used. Signals present a slight ripple due to the discrete nature of the ray tracing simulation.

According to the theoretical model developed above, a sensor with PLA plates can be built for the proposed application with the characteristics described below. Additionally, the same architecture will be tested replacing the PLA plates with thin AISI 301 Steel leaf springs, which will allow a wider lens displacement under lower loads in order to obtain a more sensitive sensor to measure smaller loads. The expected response for both studied configurations is shown in Figure 5.

- Optical assembly
  – Light source: LED Kingbright APTD1608LSECK/J3-PF (Taipei, China), Red Color.
  – Pinhole data: $x_{ph}=4$ mm, $d_{ph}=1.5$ mm, $P^{ph}\approx 7$ μWW
  – Lens data: PMMA Cylindrical lens, $x_{c}=9.5$ mm, $R_{L}=1$ mm, $n_{lens}=1.49$
  – Phodo-detector: OPR5911 Quad Photodiode, 2 × 2 Matrix. Phodo-diode size: 1.27 mm, responsitivity r=0.45 mA/mW, $x_{s}=11.5$ mm.
- Elastic frame
  – Effective plate length: L=10 mm
  – Plate width: b=10 mm
  – Plate thickness for PLA case: h=1 mm
  – Plate thickness for AISI 301 case: h=0.1 mm
  Expected range of measurement for PLA case: ±45 N
  Expected range of measurement for AISI 301 case: ±10 N

**Figure 6:**
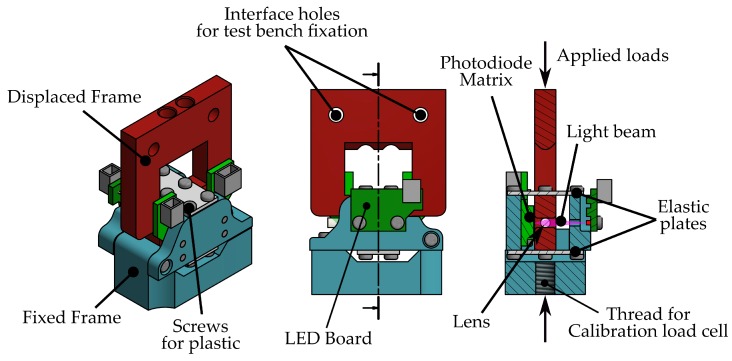
Mechanical drawings of the implemented force sensor. Blue parts are rigidly attached among them and hold the LED and Photo-Detector. The red part holds the lens and presents a slight relative displacement with respect to the blue parts when a load is applied.

The geometry of the sensor has been designed to be as similar as possible to the definitive implementation that will be integrated into the exoskeleton; however, several modifications have been performed to adapt it to the test bench that will be used for the purpose of this study. As can be seen in Figure 6, there are two different rigid frames that host the photo-electronic devices (Blue parts) and lens (Red part). These frames are attached by the two elastic plates that will allow a relative displacement in the direction of the applied loads, disturbing the light beam.

### 2.2. Signal Conditioning, Acquisition and Processing

The chosen device to measure the incident light is a quadruple (2 × 2) photo-diode matrix. This sensor features four outputs that correspond to the current that passes throughout each anode, which is proportional to the light power that receives that diode. Following the cost-effective philosophy, the signal conditioning and acquisition circuitry have been designed to use common electronic elements.

**Figure 7:**
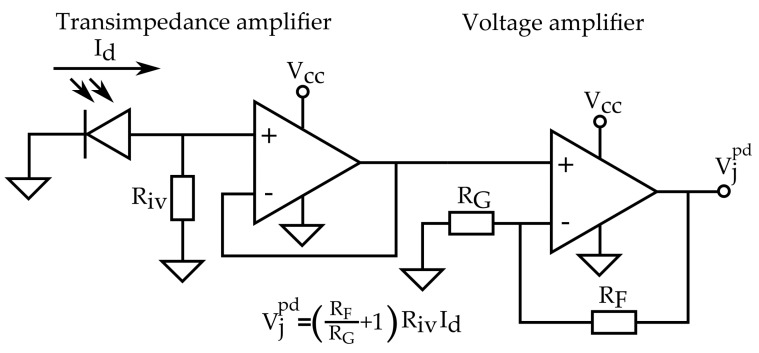
Signal conditioning circuit implemented for each individual photo-diode. The expression of the relation between output voltage ($V_{j}^{pd}$) and current in the photodiode ($I_{d}$) is shown.

In particular, for each photo-diode, the anode current is converted to a voltage value by a non-inverting transimpedance circuit using general purpose operational amplifiers. Afterwards, this voltage value is amplified by a noninverting amplifier stage, as shown in Figure 7. Since the circuit will be supplied with the 5 V ($V_{cc}$) output of a micro-controller, all operational amplifiers are asymmetrically powered. Additionally, this fact will improve the range of measurement since it is not expected (nor desired) that conditioned signals ($V_{j}^{pd}$) reach negative values.

Despite the outputs ($V_{j}^{pd}$) being combined using analogical components in order to get only one analog output, for this study, the sensor will output four voltages corresponding to each photo-diode. Output signals are read by the micro-controller throughout a multiplexed analog-to-digital converter (ADC) so that they can be added and subtracted digitally reducing the risk signal saturation. The micro-controller implements a digital filter to reduce the ripple introduced by the ADC.

For the studied implementation, the technical details can be summarized as follows:

- Operational amplifiers: 2× Quadruple general-purpose TI LM324 (One device per pair of photo-diodes).
- Resistance values: $R_{iv}=30$ kΩ, $R_{F}=120$ kΩ, $R_{G}=1.5$ kΩ.
- Resulting conversion factor: A=2.43 V/mA
- Micro-controller: ATmega1280 that features $V_{cc}=5$ V and 10 bit ADC.
- Digital filter: Mean filter with a time window of eight values.

### 2.3. Fitting Models

All the previous mathematical expressions are useful for design and sizing stages; however, the performed approximations cannot model many aspects such as color sensitivity of photo-diodes, optical misalignment and imperfection during the manufacture process or hysteresis of constructive materials. Thus, this equation cannot be used to accurately predict the performance of the proposed sensor. Hence, to deal with these effects, four fitting models will be used in order to approximate the real behavior of the sensor.

#### 2.3.1. Polynomial Fit

The most simple model of all that will be studied is a third order polynomial fit (Equation (20)) of a set of pairs voltage–force values. This approximation is expected to be able to deal with the nonlinearity of the optical model as well as reproduce the deviation from the ideal model due to optical misalignment. However, polynomials does not offer any mathematical way to model hysteresis cycles.

Despite being the less complex of the studied models, it has an advantage that can make it the most suitable solution for certain applications. Concretely, this approximation only needs a minimum of samples equal to the order of the polynomial plus one. Therefore, it can be fitted by recording the sensor response with a simple set of weights. Several samples for a certain weight can be acquired in order to use a least-square fitting method to obtain an averaged sensor curve:

(20) $$\begin{array}{c} F=\sum_{i=0}^{3}a_{i}{\left(V_{2}^{pd}-V_{1}^{pd}\right)}^{i}. \end{array}$$

#### 2.3.2. Generalized Prandtl–Ishlinksii Model

The Generalized Prandtl–Ishlinskii model [32] (GPI) is one of the most extended approximations to model systems that exhibit nonlinear, asymmetrical, and saturated hysteresis cycles [33,34,35,36]. This model represents the response of the system as a weighted sum of different play operators ($S_{r_{i}}$) as shown in Equation (21), where *V* corresponds to the output of the sensor ($V=V_{2}^{pd}-V_{1}^{pd}$), F(t) is the applied force and $p(r_{i})$ are the different weights computed as shown in Equation (23). Regarding the play operators ($S_{r_{i}}$), they are defined by two envelope functions ($\gamma_{r}$,$\gamma_{l}$ that correspond to the rising and decreasing curves of an hysteresis cycle (Equations (25) and (26)). As it can be seen in Equation (22), all the play operators have the same envelope functions but differ in the parameter $r_{i}$ that determines the width of the hysteresis loop. These width parameters are established in Equation (24). The stated GPI model presents a series of free parameters ($\alpha ,\rho ,\tau ,a_{j},b_{j}$) that can be determined by using optimization techniques from a set of samples (V(t),F(t)). The number of finite sums (*n*) can be manually stated since weight ($p(r_{i})$) rapidly will become null for higher *i* values:

(21) $$\begin{array}{cc} V(F(t)) & =\sum_{i=0}^{n}p\left(r_{i}\right)S_{r_{i}}\left[F\left(t\right)\right], \end{array}$$

(22) $$\begin{array}{cc} S_{r_{i}} & =\left\{\begin{array}{c} \max\left(\gamma_{r}-r_{i},S_{r_{i-1}}\right)\;\mathrm{for}\;\dot{F}\left(t\right)\geq 0,\\ \min\left(\gamma_{l}+r_{i},S_{r_{i-1}}\right)\;\mathrm{for}\;\dot{F}\left(t\right)<0, \end{array}\right. \end{array}$$

(23) $$\begin{array}{cc} p(r_{i}) & =\rho e^{-\tau r_{i}}, \end{array}$$

(24) $$\begin{array}{cc} r_{i} & =\alpha i, \end{array}$$

(25) $$\begin{array}{cc} \gamma_{r} & =a_{1}\tanh\left(a_{2}F\left(t\right)+a_{3}\right)+a_{4}, \end{array}$$

(26) $$\begin{array}{cc} \gamma_{l} & =b_{1}\tanh\left(b_{2}F\left(t\right)+b_{3}\right)+b_{4}. \end{array}$$

The computed model allows for computing the response of the sensor under certain load conditions. However, the desired result consists of the determination of the force that produces the measured response of the sensor (F(V(t))). Thus, the inverse model must be obtained, which can be analytically inferred from the direct model as shown in Equations (27) to (32):

(27) $$\begin{array}{ccc} F(V(t)) & =V^{-1}\left(F\left(t\right)\right)=\gamma^{-1}\left(\sum_{i=0}^{n}g_{i}R_{r_{i}}\left[V\left(t\right)\right]\right), & \end{array}$$

(28) $$\begin{array}{cc} R & =\left\{\begin{array}{c} \max\left(V\left(t\right)-q_{i},R_{r_{i-1}}\right)\;\mathrm{for}\;\dot{V}\left(t\right)\geq 0,\\ \min\left(V\left(t\right)+q_{i},S_{r_{i-1}}\right)\;\mathrm{for}\;\dot{V}\left(t\right)<0, \end{array}\right. \end{array}$$

(29) $$\begin{array}{cc} \gamma^{-1} & =\left\{\begin{array}{c} \gamma_{r}^{-1}\;\mathrm{for}\;\dot{V}\left(t\right)\geq 0,\\ \gamma_{l}^{-1}\;\mathrm{for}\;\dot{V}\left(t\right)<0, \end{array}\right. \end{array}$$

(30) $$\begin{array}{cc} g_{j} & =-\frac{p_{j}}{\left(p\left(r_{0}\right)+\sum_{i=1}^{j}p\left(r_{i}\right)\right)\left(p_{r_{0}}+\sum_{i=1}^{j-i}p\left(r_{i}\right)\right)}, \end{array}$$

(31) $$\begin{array}{cc} g_{0} & =\frac{1}{p_{r_{0}}}, \end{array}$$

(32) $$\begin{array}{cc} q_{j} & =\sum_{i=0}^{j}p\left(r_{i}\right)\left(r_{j}-r_{i}\right). \end{array}$$

#### 2.3.3. Artificial Neural Networks

Artificial Neural Networks (ANN) are a powerful tool for fitting complex models [37] and have already been successfully used in hysteresis modelling and compensation applications [38,39,40,41]. Rather than stating a series of equations with physical meaning, this solution relies on optimizing an established network of interconnected simple operators, or neurons, with a relatively big set of free parameters (at least more numerous than the alternatives exposed above).

**Figure 8:**
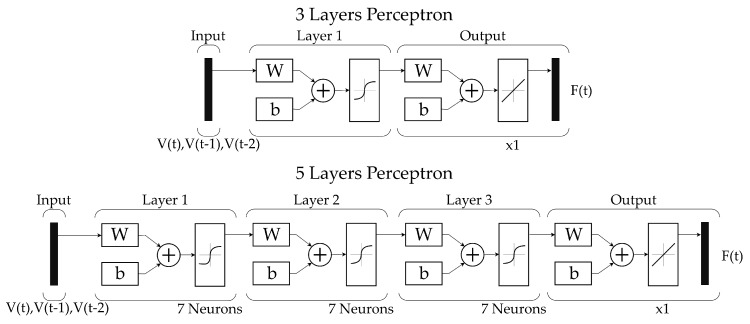
Schematic view of both 3 and 5-layer perceptrons used to model the hysteretic behaviour of the sensor. Both neural networks feature three inputs corresponding to the actual sensor voltage and the two previous values; hidden layers of seven neurons and an output layer that returns a single value, corresponding to the estimated force measurement.

For the presented application, the chosen network architecture is the Multilayer Perceptron [42]. According to Irie et al. [43], a three-layer perceptron, with a sufficient number of neurons, should be able to fit any arbitrary mapping function. Due to the foreseeable hysteretic behaviour of the sensor, the relation between the applied force (F(t)) and the measured signal of the sensor (V(t)) is not a proper mathematical function. However, if it is assumed that the estimated force may be univocally computed as a function of the current sensor measure and those taken in previous time steps ($F\left(t_{i}\right)=f(V\left(t_{i}\right),V\left(t_{i-1}\right),V\left(t_{i-2}\right)$), then Irie’s conditions are accomplished. In particular, the three-layer perceptron (3LP) that will be trained has the next characteristics (illustrated at the top of Figure 8):

- Input layer: three inputs corresponding to $V(t_{i})$, $V(t_{i-1})$, $V(t_{i-2})$ to be able to model the temporal dependency of the hysteresis.
- Hidden layer: seven neurons with symmetric sigmoid as transfer functions.
- Output layer: one output neuron with linear transfer function that returns the estimated F(t).
- Training algorithm: Levenberg–Marquardt backpropagation algorithm.

Additionally, since it is expected to obtain a reasonable amount of training data the five-layer perceptron (5LP), shown at the bottom of Figure 8, will be also trained in order to check whether it can increase the performance of the sensor or it will overfit the model.

### 2.4. Experimental Setup

The aim of the proposed experimentation is the evaluation of the performance of the new optical force sensor presented in this paper. In order to perform this assessment, a comparison of the sensor response using two different materials for the elastic plates, PLA and spring-steel, is carried out. Moreover, the different fitting algorithms, exposed in the previous section, are evaluated for both versions of the optical force sensor.

**Figure 9:**
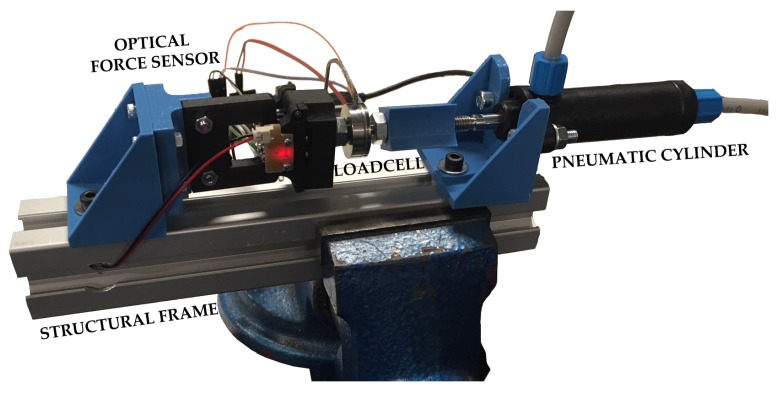
Test bench for validation experiment.

For this purpose, the test bench shown in Figure 9 has been developed. This setup consists of the following elements:

- Optical force sensor. The developed sensor that is going to be validated with the experiment.
- Commercial load cell. A calibrated and industrial grade force sensor, which is used as a reference for the fitting model procedure and validation. The selected load cell model is LCM201–100N manufactured by Omega (Stamford, CT, USA).
- Active element to apply force. A double-acting pneumatic cylinder (Festo DFK-16-40-P (Esslingen, Germany)) is used to apply the desired forces to the force sensors during the experiment. Two proportional pressure control valves (Festo MPPE-3-1/8-10-010) have been selected to control the pressure applied on the cylinder.
- Structural frame. Structure where all elements are mounted.

The experiment was conducted in two different steps. The first one is a calibration phase, where the different sensor models are computed by training the four fitting approaches exposed in the previous section. For this aim, the pneumatic cylinder is driven with the input pressure signal ($p_{input}$) exposed in Equation (33), with frequencies in the range of [0, 2] Hz and an amplitude (*A*) in the range of ±3.5 Bar for the sensor based on PLA elastic plates and ±1 Bar for the sensor based on spring-steel elastic plates. The response of the calibrated load cell is used as input for training the fitting algorithms in order to obtain the model of the optical force sensor:

(33) $$\begin{array}{c} p_{input}\left(t\right)=A\cdot sin\left(\frac{2\pi t}{127}\right)\cdot sin\left(\frac{2\pi }{0.6}\cdot sin\left(\frac{2\pi t}{9}\right)\right). \end{array}$$

Once the different sensor models are computed in the calibration phase, the second step is the validation of the these models. For this purpose, four different signals were selected to excite the optical force sensor with as much heterogeneous inputs as possible:

- 1 Hz sine wave input. Sine signal with a frequency of 1 Hz with the same maximum amplitude (*A*) previously used in the calibration phase.
- Multi-Frequency wave input. Signal composed of different sine waves in order to obtain a signal with frequency and amplitude variations.
- Random wave input. Signal with random values with a maximum frequency of 1.5 Hz and the maximum amplitude used in the calibration phase (*A*).
- Human interaction. Signal obtained by direct interaction with the test bench without using the pneumatic cylinder.

Each trained sensor model is used to obtain an estimation of the force applied to the system. This response is compared with the one obtained with the commercial load cell, which is considered the objective behavior. In the following sections, all of these results are presented and explained in detail.

## 3. Results and Discussion

### 3.1. Real Sensor Performance

**Figure 10:**
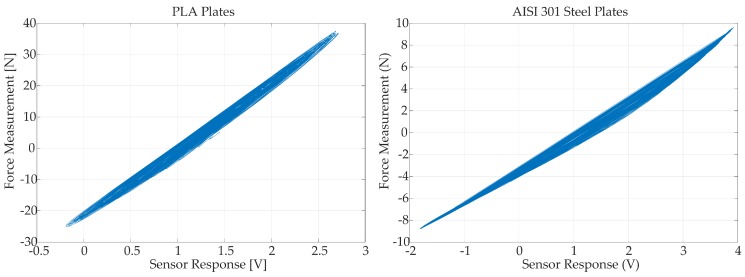
Sensor voltage output compared to the force measured in the commercial load cell, for both tested configurations. The shown graphs are the result of overlapping all the load–unload curves applied during the calibration process, revealing the shape of the hysteresis cycle for each sensor.

In Figure 10, the response of the optical force sensor prototype for both PLA and spring-steel elastic plates is presented. These responses are measured with the input signal $p_{input}$, expressed in Equation (33). In these plots, the existence of hysteresis can be appreciated for both versions of elastic plates. It must be pointed out that the hysteresis cycle of the steel based sensor is significantly asymmetrical, presumably due to the presence of bendings and residual burrs product of the machining process. In contrast, the PLA based sensor has a smoother behavior, due to a more accurate fabrication process, but with a wider hysteresis.

The comparison between the real and the theoretical performance (Figure 4) shows that the real behaviour differs from the expected sigmoid response. This fact results from the assumptions made in the optical simulations, such as homogeneous light distribution outputting from the LED source or neglected secondary reflections and refractions that can lead to a different light power distribution. In spite of this, the theoretical model gives a correct order of magnitude of the measured voltage and force.

Regarding the PLA-based sensor, the real performance shows that the optical system is heavily misaligned, resulting in a sensor response of about 1 V when no force is applied. Additionally, the sensor response covers a range of about 3 V in contrast to the 6 V that spans the theoretical model. This fact implies that the real PLA plates are almost twice as stiff as expected. This is consistent with the range of values for the elastic modulus of (2020 to 3550 MPa) established by Lanzotti et al. [30]. Another source of error in this aspect can be that the real behaviour of the elastic interfaces in somewhat between the simply supported and fixed beam approximations due to the significant thickness of the plate.

As for the steel-based implementation, the initial optical misalignment is similar to the measured one for the PLA plates. Thus, the main source of misalignment lies in the fixation of the optical elements in the common frame for both sensors rather than in the plates. In this case, the span of the range of measurement of the sensor is quite consistent with the beam-theory approximation, since the mechanical properties of this normalized material are more accurately determined. Additionally, the low thickness of the plate allows the rotation of the beam axis around the frame edge that acts like the support, resulting in a better approximation to the ideal conditions.

### 3.2. Model Fitting Performance

**Figure 11:**
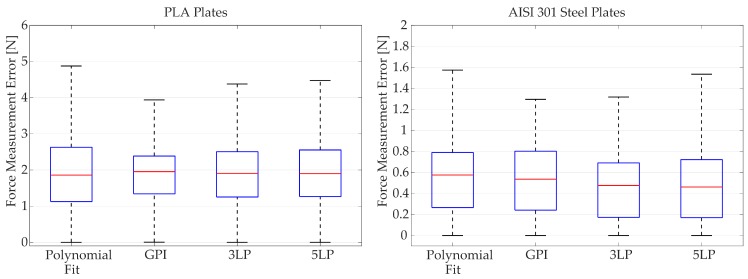
Error distribution of each fitting model evaluated over all the test signals. The red line indicates the mean value, blue boxes enclose the samples between percentiles 25 and 75, and black whiskers covers an standard deviation of ±2.7SD.

**Figure 12:**
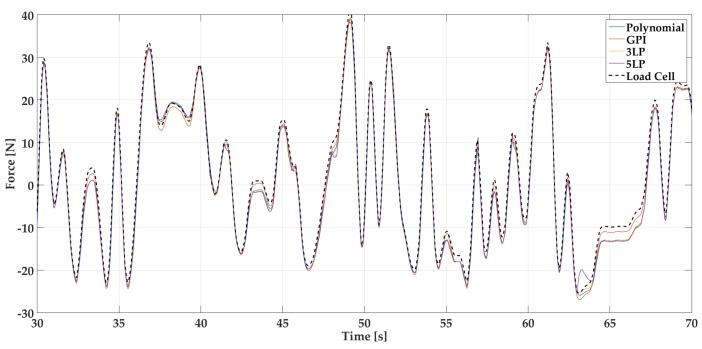
Time response of all fitting models and load cell signal for a 40 s sample of the Random Signal test for PLA based sensor.

**Figure 13:**
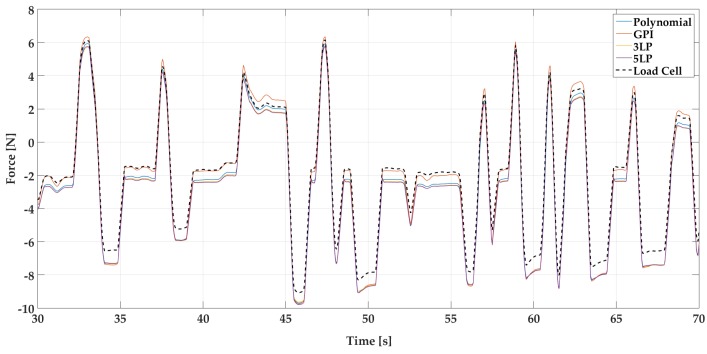
Time response of all fitting models and load cell signal for a 40 s sample of the Random Signal test for steel based sensors.

These responses are used as training data for the four proposed fitting algorithms: polynomial fit, generalized Prandtl–Ishlinskii model (GPI), 3-layer perceptron (3LP) and 5-layer perceptron (5LP). The statistical analysis of the performance for each model and signal is summarized in Table 1 and Figure 11. Additionally, Figure 12 and Figure 13 show a visual example of the performance of each model applied on a small sample of the random signal input. A series of more detailed figures that help to understand the particularities of each model can be found in Appendix A.

#### 3.2.1. Polynomial Fit Analysis

**Figure A1:**
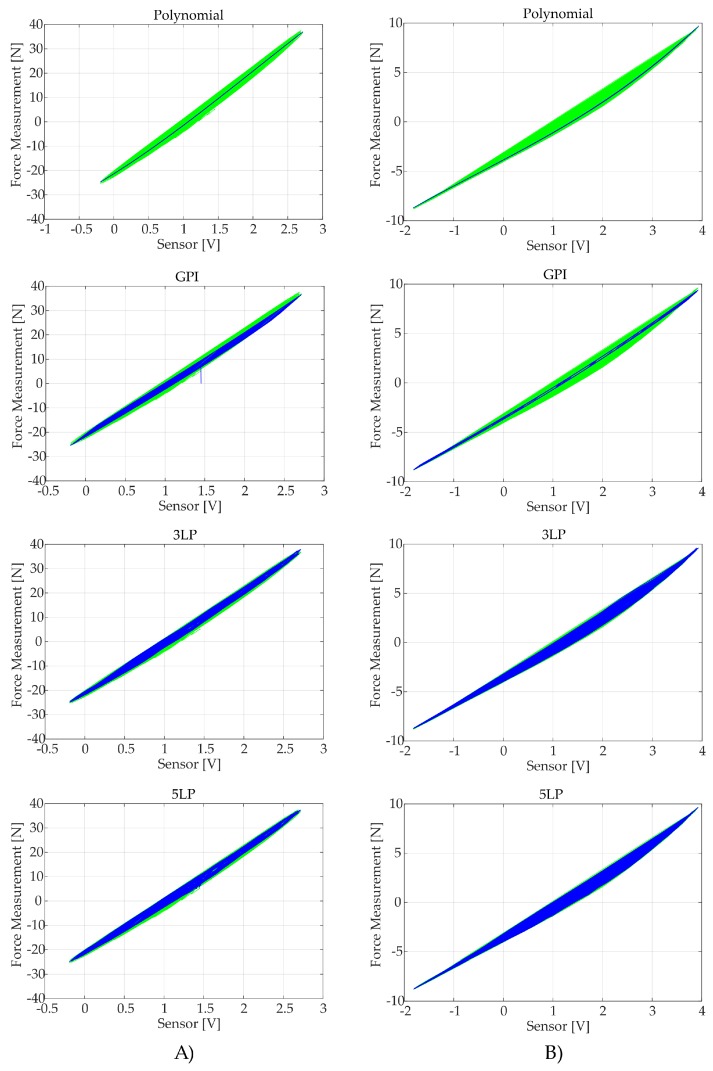
Response of each computed model (blue) superposed to the calibration data (green), where (**A**) corresponds to the data of the PLA based sensor and (**B**) to the steel based sensor.

Polynomial fit cannot model the hysteretic behaviour of the sensor, but it can fit a mean curve in order to uniformly distribute the error for all force values. As it can be seen in Figure A1, for the PLA sensor, it is able to compute a mean curve along the cycle, while in the steel sensor it does fit the lower envelope of the hysteresis.

**Figure A2:**
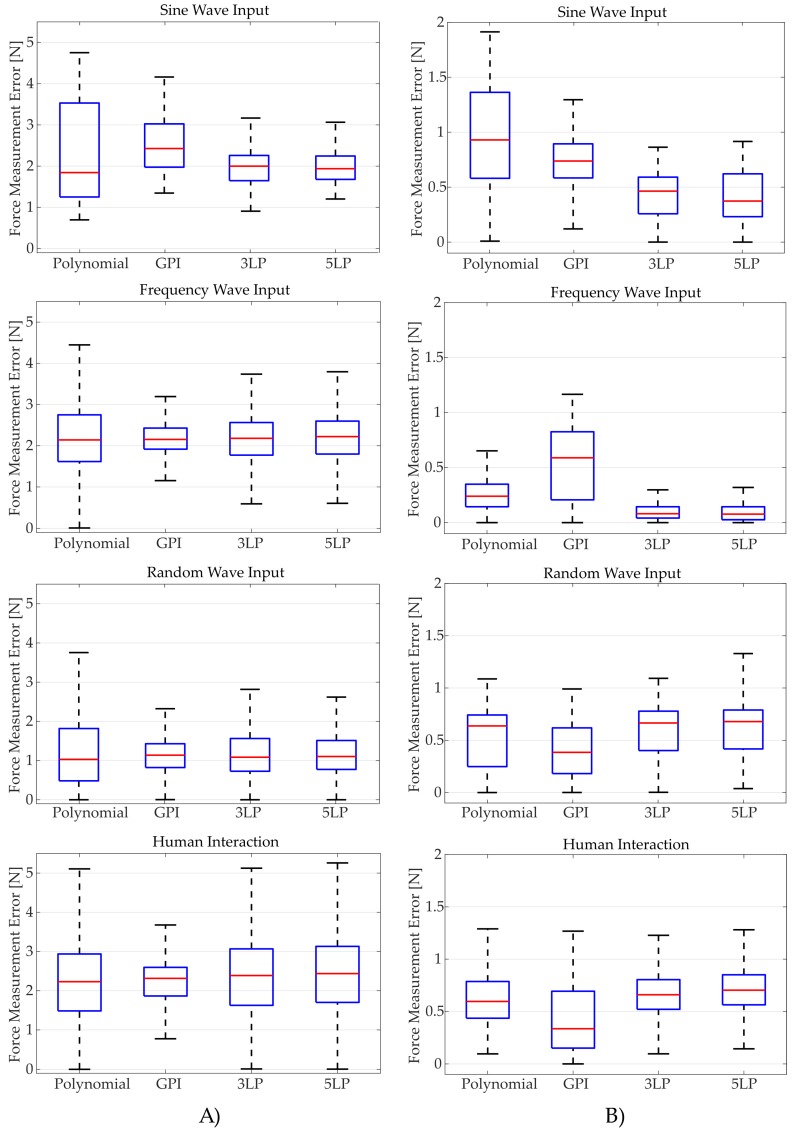
Error distribution computed for each tested kind of signal and model, where (**A**) corresponds to the data of the PLA based sensor and (**B**) to the steel based sensor.

**Figure A3:**
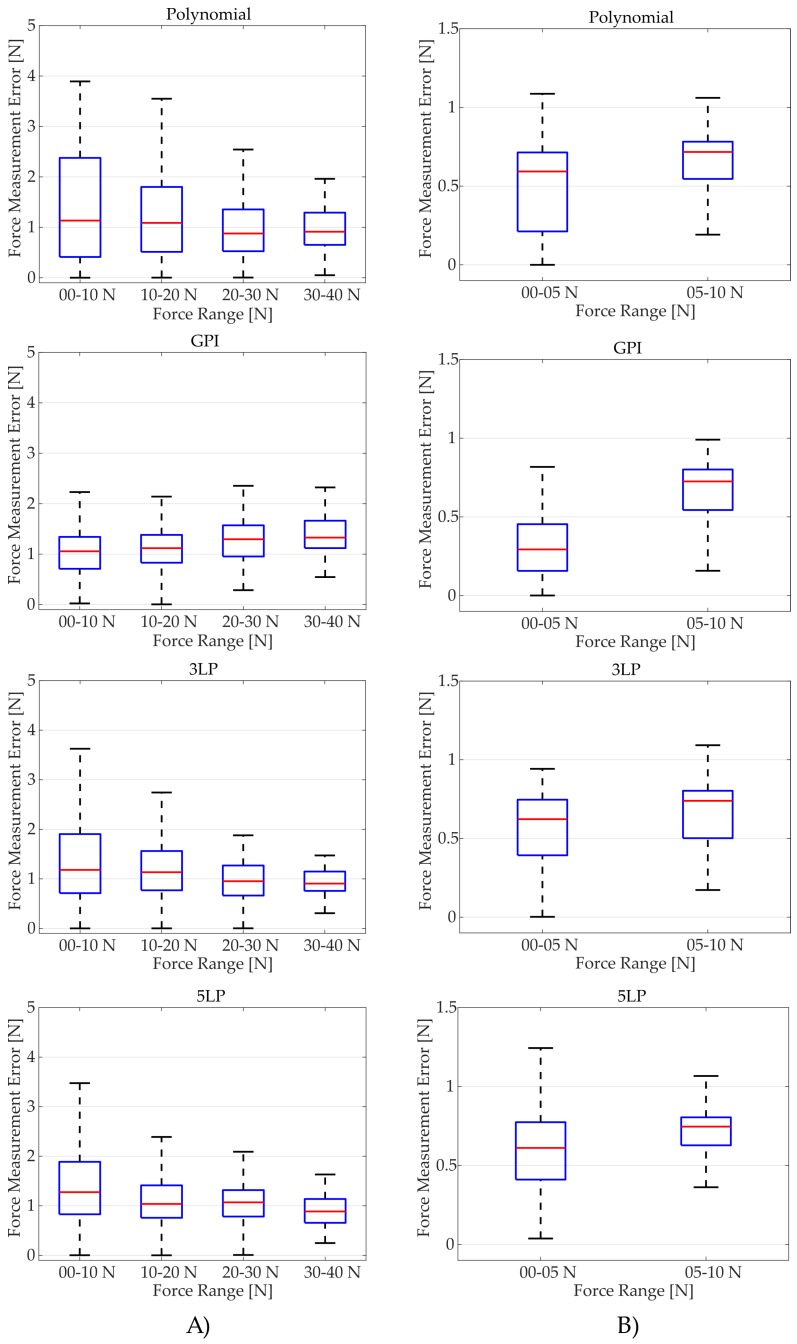
Error distribution computed for the Random Signal test and range of force. Where (**A**) corresponds to the data of the PLA based sensor and (**B**) to the steel based sensor.

This fact produces a high error dispersion for any kind of input signal, which is displayed in Figure A2. However, for the hysteresis cycle that features the PLA sensor, this error dispersion becomes closer as the force values increase and the cycle becomes narrower (Figure A3).

**Figure A4:**
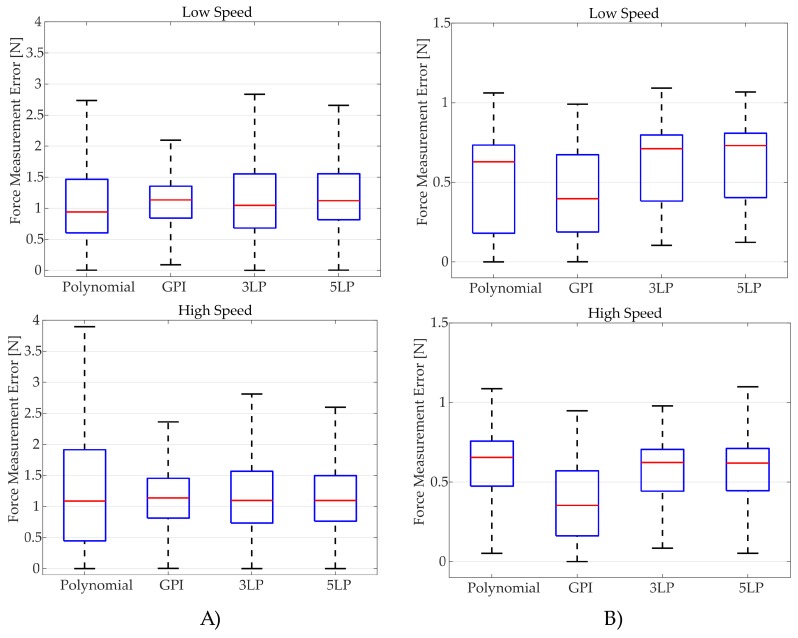
Error distribution computed for the Random Signal test and range of variation of the force. (Low Speed ≤5 N/s; High Speed >5 N/s). Where (**A**) corresponds to the data of the PLA based sensor and (**B**) to the steel based sensor.

Since this approximation does not model the dynamic behaviour of the sensor, the error is rate-dependent. Figure A4 shows a opposite behaviour depending on the shape of the hysteresis cycle: the PLA sensor features an increase in the dispersion of the error for fast variations in the force measurement, while the steel sensor presents the opposite reaction.

**Figure A5:**
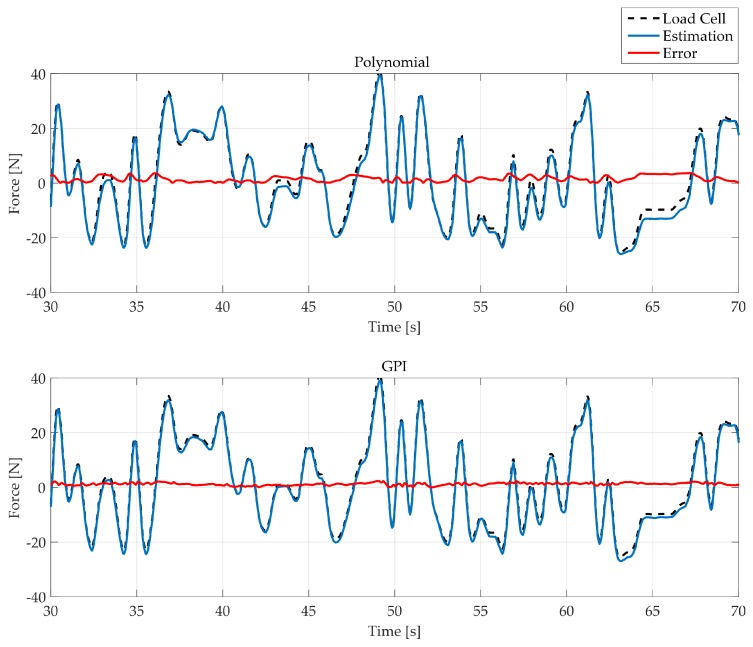

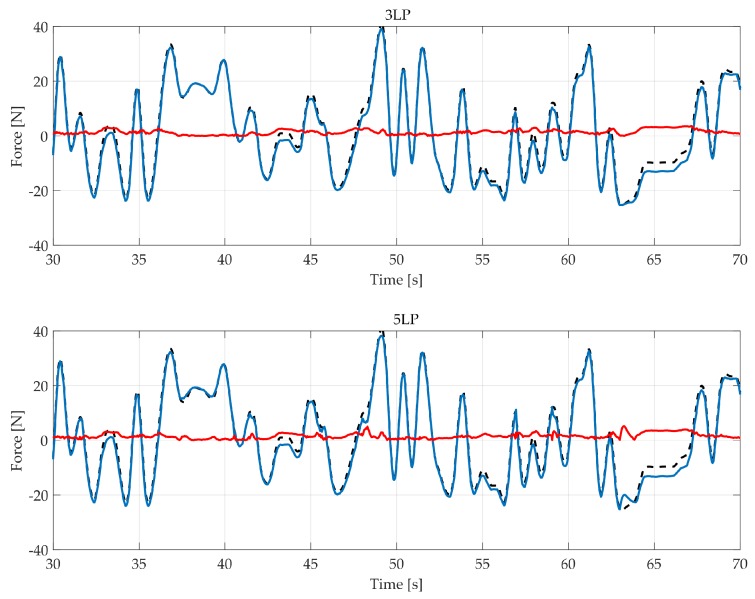
Detailed view of the time response shown in Figure 12 for each model.

**Figure A6:**
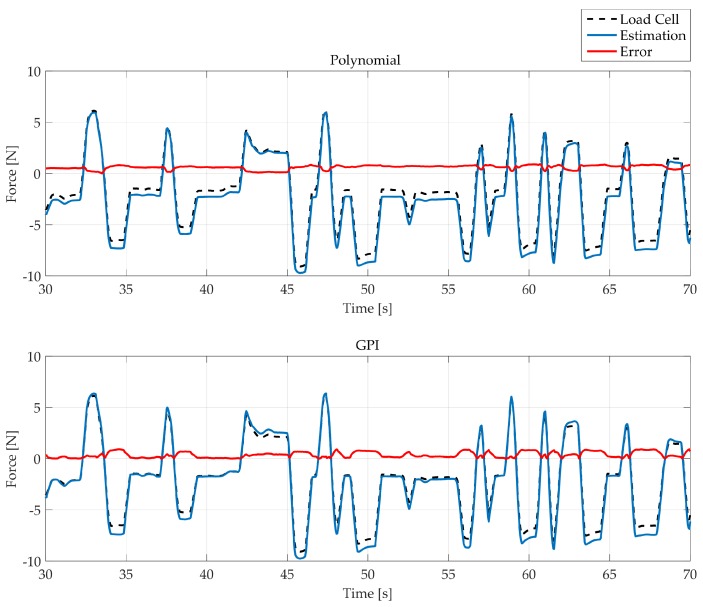

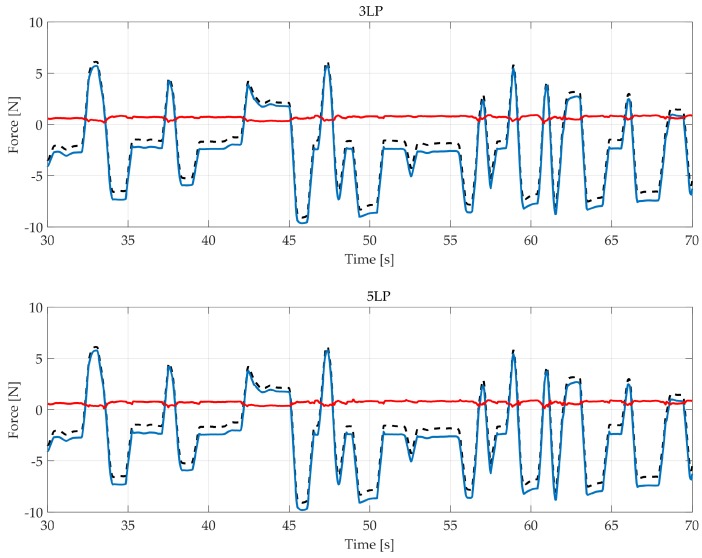
Detailed view of the time response shown in Figure 13 for each model.

Figure A5 and Figure A6 illustrate the performance of the polynomial model for an example signal. It can be seen that it outputs an estimation that has a time response quite similar to the load cell’s one. This is numerically reflected in the cross correlation coefficient (Corr Coef) of Table 1, which is almost 1.

#### 3.2.2. Generalized Prandtl–Ishlinskii Model Analysis

The generalized Prandtl–Ishlinskii model (GPI) has been able to partially model both hysteresis cycles of each kind of sensor. Figure A1 shows a quite approximate loop for the PLA sensor, but an excessively narrow response for the steel one.

In contrast to the polynomial model, this approximation is able to obtain a centered model in both cases, resulting in the tight error distributions displayed in Figure A2. For the PLA case, this model results in a quite uniform error distribution independently to the force values, while in the steel implementation it behaves similarly to the polynomial case (Figure A3) since the obtained hysteresis loop is almost negligible. For both sensor implementations, the error distribution is rate independent, as illustrated in Figure A4.

Examples shown in Figure A5 and Figure A6 reveal that this model has a quite approximate response for the PLA case, while in the steel case it tends to return higher absolute values than the load cell.

#### 3.2.3. Multilayer Perceptron Analysis

Figure A1 shows that both kinds of perceptron can model the hysteresis cycle of both sensors, resulting in an apparently more suitable response than the previous models.

Analyzing the corresponding error distributions plotted in Figure A2, it can be seen that in most cases they are comparable to the polynomial approximation, only outstanding for the “Frequency Wave Input” case, which is the most similar one to the training case. A similar trend to the polynomial approximation can be seen when evaluating the error for different force ranges (Figure A3). As for the rate dependency, they only present marginal differences for the steel case (Figure A4).

The time response represented in Figure A5 and Figure A6 reveals also a behaviour similar to the polynomial fit. Moreover, 5LP can lead to wrong dynamic evolution as it can be seen between seconds 60 and 65 in the 5LP representation of Figure A5.

#### 3.2.4. Comparative Analysis

When comparing the overall performance of each model taking into account all the analyzed signals (Figure 11), it is possible to conclude that there is not a model that stands out from the rest of the studied approximations. As shown in the last row of Table 1, all of the models have a similar mean absolute error (MAE) and median of the absolute error (MEDIAN), which are about the 5% and 6% of the maximum absolute force measurable for the PLA (45 N) and steel (10 N) sensors, respectively.

The only significant differences can be found in the dispersion of the error, quantified by the standard deviation (SD). In this regard, the GPI model produces narrower deviations than neural networks, which in turn are tighter than the polynomial fit.

As for the dynamical response of the models, they all produce signals that are quite similar to the expected one. This fact can be visually checked in Figure 12 and Figure 13 and numerically evaluated with the cross correlation coefficients (Corr Coef) of the table, which are extremely close to the unit (identical signal).

In short, all models have certain features and shortcomings that can make them more or less suitable for certain working conditions, depending on the selection of each one of the concrete applications or available material resources. The characteristics and suitability of each model can be summarized as follows:

- Polynomial fit: It is the most simple model, which does not need a complex infrastructure to be calibrated. It normally achieves a centered solution that, despite not correcting the hysteresis, can distribute the error across all measurement ranges. Additionally, due to the simplicity of the required mathematical operations, this is the fastest model to evaluate, which allows its implementation in real-time applications without requiring much computing power. This approximation may be the suitable for applications where only the dynamic evolution of the force is interesting, such as movement intention detection, endstops or coarse force controllers.
- Generalized Prandtl–Ishlinskii model: This is the most balanced model, which has enough free parameters to be able to model an hysteresis cycle while avoiding overfitting artifacts. It must be pointed out that, due to limitations of the optimization process, this alternative was trained with only the 2.5% of the acquired calibration data. Therefore, one can conclude that this model is outstanding concerning the generalization capability, being able to successfully extrapolate non-trained inputs. Its main disadvantage is the computational cost that requires its optimization. Additionally, when evaluating individual samples, it is almost 350 times slower (RCT) than the polynomial counterpart, which does not imply that it cannot be implemented for real-time applications, but it will require more computing power for the same sampling frequency. With well-chosen envelope functions, this might be the most suitable model for applications where the applied force pattern is unforeseeable, such as accurate measurements of human–machine interaction.
- Multilayer Perceptrons: Perceptrons are halfway solutions between polynomials and GPI. Like most artificial neural networks, they suffer from lack of generalization capability, so they can lead to wrong extrapolation or overfitting and attention must be paid when choosing their architecture. However, they are easy to train, require moderate computing power, and provide outstanding results when the working conditions are similar to the training ones.

## 4. Conclusions

In conclusion, the proposed sensor architecture has proven to have a coherent measurable response when an external compression–extension force is applied. By using cheap and common fabrication techniques together with the application of one of the proposed models, it is possible to obtain a sensor with a reasonable performance (in comparison with an industrial-grade load cell) that is completely customizable in force measurement and sensitivity and whose geometry can be adapted to be integrated into a device that is in the design process. Once built and integrated, this architecture allows a fast and handy modification of the desired range of measurement.

Once known the discrepancy between the theoretical model and the real response, future research lines will try to improve the approximations taking into account further effects to obtain more accurate expressions that could help to predict the behavior of the designed sensor. This refined model together with the flexibility that provides the 3D printing technologies would allow for automatically generating and building a sensor with the desired performance, thereby incorporating the benefits of the fast prototyping techniques to the force sensing technology. It is important to state that, ultimately, a fitting model would be still mandatory in order to ensure the robustness of the sensor to manufacture imperfections.

As for the measurement error that these models try to minimize, there are several strategies to explore. On one side, the use of better manufactured steel plates might lead to narrower hysteresis cycles, reducing the dependency on the fitting model and their training issues; however, this fact may increase the fabrication cost and reduce the flexibility of design. Alternatively, the use of the Generalized Prantl-Ishlinskii model is encouraging and seems to have a good generalization capability. The implemented GPI model, however, was limited to symmetrical sigmoid envelopes, which do not fit the real shape of the hysteresis well. Additionally, the optimization method used to compute this model is not the fastest and does not allow using all the available data to train the system, resulting in a loss of its potential to compensate for the error. Therefore, the implementation of a Prandtl–Ishlinskii model with asymmetrical envelopes and more specialized optimization methods, such as particle swarm optimization [44], will allow a more accurate model identification that can significantly reduce the estimation error due to insufficient hysteresis compensation.

## Figures and Tables

**Table 1:** This table contains statistical information about the results obtained in the validation experiment. MAE refers to the Mean Absolute Error, MEDIAN to the Median of the absolute error, SD to the Standard Deviation, Corr Coeff to the Cross correlation coefficient, Outliers to the percentage of samples that lie out of the MEDIAN ± 2SD interval, and RCT to the relative computation time with respect to the time required to compute the Polynomial Fit.

Input	Variable	Polynomial Fit		GPI Model		3LP		5LP	
		PLA	STEEL	PLA	STEEL	PLA	STEEL	PLA	STEEL
Sine Wave	MAE (N)	2.3147	0.9379	2.5134	0.7312	1.9853	0.4288	1.9974	0.4091
	MEDIAN (N)	1.8432	0.9299	2.4267	0.7378	1.9999	0.4639	1.9372	0.37341
	SD (N)	1.2147	0.4754	0.8571	0.2227	0.4484	0.2059	0.4209	0.2232
	Corr Coef	0.9979	0.9969	0.9991	0.9994	0.9998	0.9997	0.9998	0.9997
	Outliers (%)	0.1050	0.0253	0.1050	0.1519	0.4202	0.0506	0.7353	0.0253
Multi-Frequency Wave	MAE (N)	2.2222	0.2916	2.1924	0.5386	2.2024	0.1076	2.2551	0.1033
	MEDIAN (N)	2.1415	0.2383	2.1531	0.5879	2.1800	0.0790	2.2220	0.0753
	SD (N)	0.9141	0.2490	0.4228	0.3327	0.6876	0.0985	0.7718	0.1130
	Corr Coef	0.9972	0.9948	0.9994	0.9927	0.9985	0.9993	0.9970	0.9992
	Outliers (%)	1.1416	9.3394	2.4099	0.0506	4.3379	5.5176	5.7585	4.6064
Random Wave	MAE (N)	1.2371	0.5313	1.1349	0.4083	1.1916	0.6022	1.2634	0.6155
	MEDIAN (N)	1.0303	0.6371	1.1378	0.3845	1.0863	0.6653	1.1042	0.6788
	SD (N)	0.9150	0.2740	0.4989	0.2593	0.6863	0.2144	0.8307	0.2179
	Corr Coef	0.9976	0.9992	0.9995	0.9988	0.9990	0.9994	0.9980	0.9995
	Outliers (%)	0.0761	0.0253	0.1015	0.0253	2.8412	0.0253	6.7986	0.0253
Human Interaction	MAE (N)	2.2618	0.6221	2.2643	0.4325	2.4103	0.6754	2.4798	0.7165
	MEDIAN (N)	2.2304	0.5953	2.3151	0.3345	2.3873	0.6594	2.4370	0.7032
	SD (N)	1.0011	0.2475	0.7065	0.3425	0.9520	0.2239	1.0353	0.2043
	Corr Coef	0.9966	0.9991	0.9988	0.9979	0.9973	0.9989	0.9960	0.9995
	Outliers (%)	0.4225	0.0253	4.1127	0.0253	0.1690	1.8983	0.4225	0.2278
Overall Statistics	RCT	1.0		345.87		109.56		137.56	
	MAE (N)	1.9271	0.5958	1.9011	0.5276	1.9236	0.4535	1.9841	0.4611
	MEDIAN (N)	1.8601	0.5753	2.0568	0.5359	2.1305	0.4762	2.2193	0.4608
	SD (N)	1.0751	0.3995	0.7849	0.32001	0.9160	0.2915	0.9945	0.3048
	Corr Coef	0.9964	0.9956	0.9984	0.9945	0.9977	0.9985	0.9967	0.9985
	Outliers (%)	0.2341	2.4487	0.6378	0.03164	0.7831	0.0063	1.6712	0.0190
